# Medium- and Long-Term Lead Stability and Echocardiographic Outcomes of Left Bundle Branch Area Pacing Compared to Right Ventricular Pacing

**DOI:** 10.3390/jcdd8120168

**Published:** 2021-11-30

**Authors:** Haojie Zhu, Zhao Wang, Xiaofei Li, Yan Yao, Zhimin Liu, Xiaohan Fan

**Affiliations:** State Key Laboratory of Cardiovascular Disease, Cardiac Arrhythmia Center, Fuwai Hospital, National Center for Cardiovascular Diseases, Chinese Academy of Medical Sciences and Peking Union Medical College, Beijing 100037, China; zhuhaojie@fuwai.com (H.Z.); wangzhao@fuwai.com (Z.W.); lixiaofei0103@163.com (X.L.); ianyao@263.net.cn (Y.Y.); liucory@163.com (Z.L.)

**Keywords:** left bundle branch area pacing, right ventricular pacing, lead stability

## Abstract

The long-term lead stability and echocardiographic outcomes of left bundle branch area pacing (LBBAP) are not fully understood. This study aimed to observe the mid-long-term clinical impact of LBBAP compared to right ventricular pacing (RVP). Consecutive bradycardia patients undergoing LBBAP or RVP were enrolled. Pacing and electrophysiological characteristics, echocardiographic measurements, and procedural complications were prospectively recorded at baseline and follow-up. LBBAP was successful in 376 of 406 patients (92.6%), while 313 patients received RVP. During a mean follow-up of 13.6 ± 7.8 months, LBBAP presented with similar pacing parameters and complications to RVP, except a significantly narrower paced QRS duration (115.7 ± 12.3 ms vs. 148.0 ± 18.0 ms, *p* < 0.001). In 228 patients with ventricular pacing burden >40%, LBBAP at last follow-up resulted in decreased left atrial diameter (LAD) (40.1 ± 8.5 mm vs. 38.5 ± 8.0 mm, *p* < 0.001) while RVP produced decreased left ventricular ejection fraction (62.7 ± 4.8% vs. 60.5 ± 6.9%, *p* < 0.001) when compared to baseline. After adjusting for age, the presence of atrial fibrillation, and other clinical factors, LBBAP was still associated with a decrease in LAD (−1.601, 95% CI −3.094–−0.109, *p* = 0.036). We conclude that LBBAP might result in more preserved echocardiographic outcomes than RVP.

## 1. Introduction

Left bundle branch area pacing (LBBAP), first reported by Huang et al. [1], has emerged as a physiological pacing technique alternative to His bundle pacing (HBP) with stable and low capture threshold and high R wave amplitude [2]. However, the long-term stability of LBBAP has not been fully understood. Traditional right ventricular pacing (RVP) is a well-established pacing strategy, but it can cause electromechanical desynchrony and significantly increase the risk of heart failure and mortality in patients with a high burden of ventricular pacing [3,4]. For LBBAP, the lead stability is an essential concern because the pacing lead needs to be deeply rotated into the interventricular septum to capture the left bundle branch (LBB) [5]. The lead performance might be interfered by continuous myocardial contraction. Recently, a large single-center cohort study demonstrated the long-term safety and feasibility of LBBAP in patients with symptomatic bradycardia or advanced heart failure [6]. However, few data are available regarding comparisons of lead stability and clinical outcomes between LBBAP and RVP. Chen et al. reported comparisons of the mid-long-term feasibility and safety between LBBAP and RVP, but echocardiographic outcomes were not analyzed during follow-up [7]. The present study aimed to compare the lead stability and echocardiographic outcomes between LBBAP and RVP during mid-long-term follow-up.

## 2. Materials and Methods

### 2.1. Study Populations

Consecutive patients receiving LBBAP or RVP procedures for symptomatic bradycardia were prospectively enrolled at Fuwai Hospital since 2019. All patients were indicated for pacemaker implantation per the American College of Cardiology, American Heart Association, and Heart Rhythm Society guidelines [8]. Patients were excluded when one or more of the following criteria was met: (1) younger than 18 years old; (2) indicated for cardiac resynchronization therapy or implantable cardioverter-defibrillator; (3) undergoing pacemaker replacement or upgrade with existing leads. All participants provided written informed consent, and the Institutional Review Board of Fuwai Hospital approved this study.

### 2.2. Procedures

LBBAP was performed by using SelectSecure pacing lead (model 3830, 69 cm, Medtronic Inc., Minneapolis, MN, USA) and a fixed-curve sheath (C315HIS, Medtronic Inc.) as previously published [5,9]. An electrophysiology recording system (Bard/Boston Scientific, Lowell, MA, USA) was used to monitor and record the intracardiac electrogram (IEGM). The “single lead method” is routinely used for LBBAP lead implant, which is similar to the “simplified nine-partition method” [10,11]. The target screwing site was identified by anatomical location and pacing mapping. Briefly, the 3830 lead was directly advanced to the RV septal area about 1.5–2.0 cm from tricuspid annulus without His mapping under RAO 30°, and pacing mapping was performed to identify a screwing site with a W-shape paced QRS morphology in the lead V1. Then, the lead was quickly screwed into the septum until premature ventricular beat with RBBB pattern was observed or the lead was seen penetrating into the septum. A pacing test was performed to confirm the capture of LBB. His mapping, unnecessary pacing tests, and repeated fluoroscopy under LAO 45° for verifying lead position were omitted to save procedure time. In most cases, the whole procedure was performed under RAO 30°. When the 3830 lead could not be screwed into the septum at the first attempt, the target site was changed to find another screwing site with suitable R wave sensing amplitude. His mapping, dual-lead technique, and contrast-enhanced image-guided method might be used in some challenging cases. During the procedure, pacing tests were performed, and the surface 12-lead ECG, IEGM, and fluoroscopy imaging were simultaneously monitored. LBB potential and potential to ventricle interval (P-V interval) were recorded. Pacing stimulus to left ventricular activation time (Sti-LVAT) in lead V5 or V6 was measured at low (at 2 V/0.4 ms) and high (at 5 V/0.4 ms) outputs. Successful LBBAP was confirmed per the previously published criteria [5,12]: (a) paced QRS morphology presented with an RBBB pattern; (b) Sti-LVAT shortened abruptly and remained shortest and constant at different testing outputs. Selective LBBAP was identified if a discrete component was present between the spike and the QRS onset on IEGM at a low output, or LBB potential could be recorded, or a transition of QRS morphology from “Qr” or “QR” type to “rsR” type could be observed in lead V1 when decreasing unipolar outputs. If LBBAP failed after 5 attempts or fluoroscopy duration exceeded 20 min, the lead was then positioned in the mid-LV septum, namely LV septal pacing (LVSP), to achieve a relatively narrow QRSd. ECG parameters were measured at a sweep speed of 100 mm/s, including P-V interval, Sti-LVAT, and paced QRS duration (pQRSd). The procedure and fluoroscopy duration for LBBAP lead implantation were recorded from the advancement of the C315 His sheath to the end of successful 3830 lead placement. Transient RBB injury was defined as new-onset RBBB during the procedure which quickly recovered after the procedure or before discharge. In contrast, persistent RBB injury referred to sustained RBBB after discharge and during follow-up.

RVP was performed with the active fixation lead positioned at the RV septum. Fluoroscopic radiographs from 45° left anterior oblique were used to confirm the lead position. The procedure and fluoroscopy duration for RVP lead were recorded from the advancement of the sheath to the end of successful implant of active-fixation pacing lead.

### 2.3. Follow-Up and Echocardiographic Evaluation

Patients were followed up with at 3 months, 6 months, 12 months, and 24 months after implant. Pacing parameters (capture threshold, impedance, sensing amplitude, percentage of ventricular pacing, pQRSd) were recorded and compared between LBBAP and RVP. Echocardiography was performed at baseline, 6 months, 12 months, and 24 months after the procedure by using Vivid E9 systems (GE Vingmed Ultrasound AS, Horten, Norway) to evaluate left ventricular end-diastolic diameter (LVEDD) and left atrial diameter (LAD). Left ventricular ejection fraction (LVEF) was measured using biplane Simpson’s method in two-dimensional transthoracic echocardiography. Device-related complications were continuously tracked, including lead dislodgement, lead perforation, pacing system infection, and other procedure-related complications. 

### 2.4. Statistical Analysis 

Continuous variables are presented as mean ± SD or median with interquartile range and compared using Student’s *t*-test or the Mann–Whitney U-test. Categorical variables are reported as numbers and percentages and compared using chi-square or Fisher’s exact test. Paired *t*-tests were used to compare data at baseline and follow-up. Multiple linear regression analysis was used to investigate the impact of LBBAP and other clinical factors on LAD. A *p*-value of <0.05 indicates statistical significance. R version 3.5.1 (R Foundation for Statistical Computing, Vienna, Austria) was used to perform all analyses.

## 3. Results

### 3.1. Baseline Characteristics

A total of 406 patients underwent LBBAP procedures, and 313 patients received RVP. Table 1 shows the comparison of baseline characteristics between patients with LBBAP and RVP. No significant differences were observed in age, gender, previous medical history, heart rates, QRS duration, echocardiographic parameters, and medications between the two groups (all *p* > 0.05). Compared with RVP, LBBAP was attempted more often in patients with RBBB or LBBB and patients with atrioventricular block (AVB) (all *p* < 0.001).

### 3.2. Procedural and Electrophysiological Parameters

LBBAP was successfully achieved in 92.6% (376/406) of patients. The remaining 30 patients who failed LBBAP finally underwent LVSP with the lead located in the deep ventricular septum. LBB potential was present in 68.1% of patients with an average P-V interval of 27.7 ± 4.7 ms. The mean Sti-LVAT at the high output was 73.9 ± 13.4 ms, similar to that at low output. During the procedure, ring capture at 2 V/0.4 ms was achieved in 97.3% of patients, with a mean threshold of 1.04 ± 0.65 V/0.4 ms. LBBAP produced a significantly narrower pQRSd than RVP did (114 ± 10.7 ms vs. 148 ± 18.0 ms). As shown in Table 2, there was no significant difference in pacing characteristics between the two groups, including capture threshold, impedance, and R wave amplitude (all *p* > 0.05). The median procedural duration for 3830 lead implantation was longer than that in RVP (11.0 min vs. 6.7 min, *p* < 0.001), as was the fluoroscopy time (5.0 min vs. 2.8 min, *p* < 0.001).

### 3.3. Pacing Parameters and Lead Stability during Follow-Up

**Figure 1:**
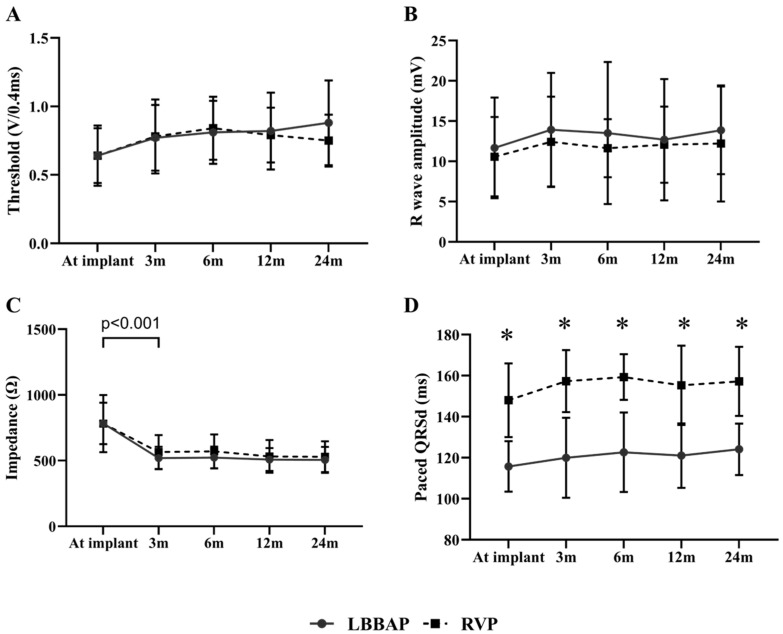
Comparison of pacing parameters between LBBAP and RVP at baseline and during follow-up. (**A**,**B**) LBBAP (solid line) produced stable capture thresholds and R wave sensing amplitudes comparable to RVP (dashed line) at baseline and during follow-up; (**C**) Both groups demonstrated a significantly decreased pacing impedance three months post-implant (*p* < 0.001) and then remained stable during follow-up; (**D**) LBBAP presented with a narrowed paced QRS duration than RVP did. The difference in QRS duration persisted between two groups during follow-up (*p* < 0.001); LBBAP = left bundle branch area pacing; RVP = right ventricular pacing.

Figure 1 Illustrates the changing trends of lead parameters during a mean follow-up of 13.6 ± 7.8 months in patients with LBBAP or RVP. The capture threshold of LBBAP was similar to that of RVP at implant (0.64 ± 0.22 V/0.4 ms vs. 0.64 ± 0.20 V/0.4 ms, *p* > 0.05) and remained stable during follow-up (Figure 1A). There were no significant differences between LBBAP and RVP in R wave amplitude and pacing impedance at baseline and during follow-up (all *p* > 0.05*) (*Figure 1B,C). However, both groups presented with a markedly decreased pacing impedance three months post-implant (both *p* < 0.001) and remained stable during follow-up. LBBAP produced a significantly narrower pQRSd than RVP (115.7 ± 12.3 ms vs. 148.0 ± 18.0 ms, *p* < 0.001), and the difference in pQRSd persisted during follow-up (Figure 1D).

### 3.4. Echocardiographic Outcomes during Follow-Up

**Figure 2:**
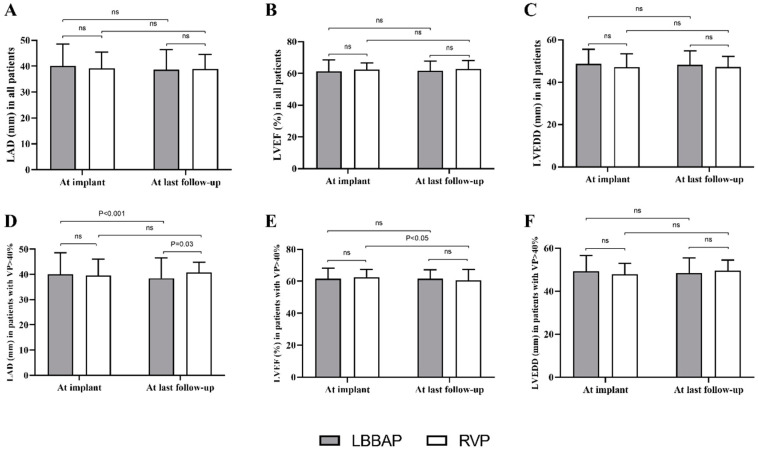
Echocardiographic measurements between LBBAP and RVP at baseline and during follow-up. (**A**–**C**) There were no significant difference in cardiac structure and function between LBBAP and RVP group during follow-up (all *p* > 0.05); (**D**–**F**) Among patients with VP% > 40%, decreased LAD (40.1 ± 8.5 mm at implant vs 38.5 ± 8.0 mm at last follow-up, *p* < 0.001) and LVEF (62.7 ± 4.8% at implant vs 60.5 ± 6.9% at last follow-up, *p* < 0.001) were observed in LBBAP group and RVP group, respectively; ns = non-significant; LBBAP = left bundle branch area pacing; RVP = right ventricular pacing; LAD = left atrial diameter; LVEF = left ventricular ejection fraction.

Echocardiographic parameters did not present a significant difference between LBBAP and RVP during the mean follow-up of 13.6 ± 7.8 months in the total study population (Figure 2A–C). Subgroup analysis stratified by VP% illustrated that in 228 patients with VP ≥ 40% (Figure 2D–F), LBBAP (*n* = 169) resulted in a significantly decreased LAD (40.1 ± 8.5 mm at implant vs. 38.5 ± 8.0 mm at last follow-up, *p* < 0.001) while no effect of RVP (*n* = 59) on LAD was observed (39.6 ± 6.5 mm vs. 40.8 ± 3.9 mm *p* > 0.05). The comparison of LAD did not differ significantly at implant between LBBAP and RVP groups. However, the mean LAD at last follow-up in patients with RVP was significantly larger than that in the LBBAP group (40.8 ± 3.9 mm vs. 38.5 ± 8.0 mm, *p* < 0.001) (Figure 2D). In addition, the mean LVEF in patients with RVP was significantly decreased (from 62.7 ± 4.8% at implant to 60.5 ± 6.9% at last follow-up, *p* < 0.001), while LBBAP resulted in a stable LVEF (from 61.6 ± 6.7% at implant to 61.6 ± 5.7% at last follow-up, *p* > 0.05) in patients with VP ≥ 40% (Figure 2E). Moderate or severe mitral regurgitation at last follow-up was not significantly different between LBBAP and RVP (8.3% vs. 9.1%, *p* > 0.05), nor was the tricuspid regurgitation (9.8% vs. 10.9%, *p* > 0.05).

Multiple linear regression analysis for the impact of LBBAP on the change of LAD (ΔLAD) are shown in Table 3. After adjusting for age, the presence of atrial fibrillation or valvular heart disease, baseline LAD and LVEF, medication and other potential clinical factors, LBBAP was still associated with a significantly negative change in ΔLAD (−1.601, 95% CI −3.094–−0.109, *p* = 0.036) when compared with RVP. In addition, baseline LAD and LVEF were also correlated with the negative change of ΔLAD (both *p* < 0.05). Atrial fibrillation was an independent risk factor for the enlarged ΔLAD (2.113, 95% CI 0.900–3.325, *p* = 0.001).

### 3.5. Procedure-Related Complications during Follow-Up

Lead dislodgement requiring lead revision occurred in one patient with LBBAP and two patients with RVP soon after the implantation procedure. One patient suffered from lead perforation after LBBAP procedure and had no symptoms except loss of pacing capture. All lead revision was successfully performed with no further symptoms or signs. RBB injury occurred in 30 of 376 patients (8.0%) and persisted in eight patients (2.1%) before discharge. During the follow-up, another two patients with RVP suffered lead dislodgement one month after the procedure and received successful ventricular lead repositioning. One patient with LVSP suffered an intermittently increased pacing threshold up to 4.0 V/0.4 ms at three months post-procedure due to severe septal fibrosis. Other device-related complications in both groups are summarized in Table 4, including pacing system infection, pocket hematoma, and pneumothorax/hemothorax.

## 4. Discussion

This single-center prospective study demonstrated the mid-long-term lead stability and echocardiographic effect of LBBAP compared with RVP. The main findings are as follows: (1) LBBAP demonstrated favorable lead performance and pacing parameters similar to RVP during mid-long-term follow-up; (2) LBBAP resulted in significantly narrower QRSd, reduced LAD, and preserved LVEF in patients with VP ≥ 40% compared with RVP; (3) lead-related complications of LBBAP were low and similar to that of RVP. Our results provide evidence for mid-long-term lead stability and safety of LBBAP, and the potential effect of LBBAP on cardiac reverse remodeling compared with RVP.

Data on LBBAP are limited due to the small sample size and short-term follow-up in most previous studies. Long-term lead stability is a significant concern for LBBAP, which might be affected by continuous myocardial contraction due to the deeply screwed LBBAP lead into the interventricular septum to capture the LBB area. Recently, Huang et al. reported the largest cohort of patients with LBBAP to date [6], and the pacing parameters of LBBAP remained stable during a mean follow-up of 18 months. Our study also demonstrated stable pacing parameters of LBBAP in most patients during follow-up, including slightly increased pacing threshold and sensing R wave amplitude and rapidly decreased impedance. Chen et al. first compared the pacing parameters between LBBAP and RVP at implant and at 18 months follow-up [7]. Our results showed slightly different findings comparing the mid-long-term lead stability between LBBAP and RVP. In their study, LBBAP pacing thresholds at implant were significantly lower than RVP and then increased gradually to a mean value similar to RVP at 18-month follow-up. However, our study did not find significant differences between LBBAP and RVP in pacing thresholds, sensing R wave amplitude, and pacing impedance at implant and each visit of post-procedure follow-up.

Moreover, our study provided comparisons of procedure duration and fluoroscopy time between LBBAP and RVP. The mean procedure duration and fluoroscopy time of LBBAP were slightly longer but close to RVP in our study. Therefore, our results, together with previous studies, manifested the reliable pacing parameters of LBBAP similar to RVP in most patients requiring ventricular pacing.

In our study, when patients were further stratified by VP%, a significantly decreased LVEF was found in patients receiving RVP with a high ventricular pacing burden. The deleterious effect of RVP on cardiac function and new-onset atrial fibrillation (AF) is widely known. Compared with RV apex pacing, HBP could result in a more physiological LV electromechanical activation and, consequently, better LA function [13] and is associated with a lower risk of AF occurrence [14]. Recently, a study compared the effect of different pacing modalities on left atrial function seven days after the procedure by using speckle-tracking echocardiography [15]. Researchers found that the absolute values of left atrial strain and strain rate increased in pacing-dependent patients with LBBAP but decreased in RVP. Our study found that LBBAP significantly resulted in reduced LAD in patients with a VP% > 40% compared with RVP during mid-long-term follow-up. The significant association between LBBAP and negative change of ΔLAD after multiple linear regression analysis indicates the undeniable effect of LBBAP on LA reverse remodeling. Other clinical factors that exerted different effect on changes of ΔLAD might be explained by follow-up duration and severity of disease. The beneficial effect of LBBAP on LAD and left atrial function might be associated with the left ventricular electromechanical synchrony induced by conduction system pacing. Whether LBBAP affects LA reverse remodeling and incidence of AF needs to be explored in future long-term studies.

Lead-related complications have been reported to be low [16], including postoperative septum perforation, postoperative lead dislodgement, intraoperative septum injury, and intraoperative lead fracture. Intraoperative lead perforation has been reported not to cause further damage if no injury of the ventricular septum is identified [6,9,17]. Some studies [6,16] reported very low occurrence of postoperative septum perforation (0.33%) and lead dislodgement (0.33%). Consistent with these findings, our previous study reported one septum perforation and one lead dislodgement within 2 h after the procedure, which occurred during the initial stage of performing LBBAP. Additionally, no postoperative septum perforation or lead dislodgement was observed in this cohort, including LBBAP cases since 2019 (*n* = 376). Moreover, transient or persistent RBB injury may occur during the LBBAP procedure. Huang et al. reported that transient RBB injury occurred in 20.4% of patients while persistent RBB injury occurred in 8.9% of patients [6]. Our group observed a relatively lower incidence of transient and persistent RBB injury (8.0% and 2.1%, respectively), which might be explained by our LBBAP procedure without distal His mapping. The LBBAP lead in our group was usually positioned by using anatomical location and pacing mapping, in which the lead screwing sites might be farther away from the RBB area. Our previous study found that 7.3% of patients with HBP were subjected to increased pacing threshold of >3.0 V/0.4 ms compared with none in patients with LBBAP [2]. The present study also showed that patients with successful LBBAP were not found to suffer from an increased LBB capture threshold >2 V/0.4 ms during a mean follow-up of 13.6 ± 7.8 months. However, loss of ventricular capture due to a high threshold of 4 V/0.4 ms was observed in one patient with LVSP at three months follow-up. This patient failed LBBAP after five attempts due to severe septal fibrosis. Finally, LVSP was performed to achieve a relatively narrow QRS duration of 126 ms with a threshold of 1.0 V/0.4 ms during the procedure. Therefore, septal fibrosis may cause unsuccessful LBBAP perioperatively and markedly increased ventricular pacing threshold in follow-up post-implant.

Several limitations need to be mentioned. Firstly, the non-randomized design is the main limitation of this single-center observational study, which necessitates cautious interpretation of our results. Individual option of pacing strategy was based on patients’ choice and physicians’ experience on device implantation. Moreover, although statistically significant differences were observed between the two groups regarding echocardiographic parameters, their magnitude and clinical significance seem limited. Outcomes with more than two years’ follow-up may provide more worthwhile data. Finally, our analysis included only bradycardia patients requiring ventricular pacing, while patients with heart failure were excluded from our study. LBBAP might play a more significant role in patients with heart failure and who require ventricular pacing. Large-scale, multicenter randomized controlled trials would provide robust evidence for the clinical application of LBBAP. If a leadless pacemaker with a suitable helix could be screwed into the ventricular septal and could capture the left bundle branch, the physiological pacing might be achieved without affecting the tricuspid function and pocket-related complications.

## 5. Conclusions

LBBAP could produce stable pacing parameters and few lead-related complications comparable with RVP during mid-long-term follow-up. Compared with RVP, LBBAP may have a beneficial effect on LA function by reducing LAD in patients with a high ventricular pacing burden.

## Figures and Tables

**Table 1:** Baseline clinical features of patients attempting LBBAP and RVP.

Variables	LBBAP (*n* = 406)	RVP (*n* = 313)	*p* Value
Age, years	64.9 ± 14.3	67.5 ± 12.2	0.080
Male, n (%)	197 (48.5%)	150 (47.9%)	0.554
Hypertension, n (%)	244 (60.1%)	200 (63.9%)	0.329
Diabetes, n (%)	79 (19.5%)	72 (23.0%)	0.292
Atrial fibrillation, n (%)	178 (43.8%)	129 (41.2%)	0.534
CAD, n (%)	76 (18.7%)	66 (21.1%)	0.470
Valvular heart disease, n (%)	35 (8.6%)	24 (7.7%)	0.748
Baseline Electrocardiogram			
Heart rate, bpm	54.7 ± 17.5	61.1 ± 17.0	0.236
QRS duration, ms	112.4 ± 24.1	98.0 ± 18.3	0.405
LBBB, n (%)	43 (10.5%)	1 (0.3%)	<0.001
RBBB, n (%)	95 (23.4%)	14 (4.5%)	<0.001
Baseline Echocardiography			
LAD, mm	40.2 ± 8.45	39.1 ± 6.30	0.060
LVEDD, mm	48.6 ± 6.91	47.1 ± 6.25	0.224
LVEF, mm	61.2 ± 7.27	62.5 ± 4.14	0.203
IVS, mm	9.8 ± 1.93	10.4 ± 4.27	0.360
Moderate or severe MR, n (%)	40 (9.9%)	28 (8.9%)	0.702
Moderate or severe TR, n (%)	38 (9.4%)	32 (10.2%)	0.705
Pacing indications			<0.001
AVB, n (%)	245 (60.3%)	86 (27.5%)	
SND, n (%)	161 (39.7%)	227 (72.5%)	
Type of device			<0.001
Double-chamber PM, n (%)	341(84.0%)	297 (94.9%)	
Single-chamber PM, n (%)	65 (16.0%)	16 (5.1%)	
Medications			
Beta blockers, n (%)	41 (10.1%)	30 (9.6%)	0.819
ACEI/ARBs, n (%)	188 (46.3%)	157 (50.2%)	0.305
CCB, n (%)	221 (54.4%)	184 (58.8%)	0.243
Antiarrhythmic drugs *, n (%)	107 (26.4%)	72 (23.0%)	0.303
NOACs, n (%)	26 (6.4%)	20 (6.4%)	0.560
Warfarin, n (%)	31 (7.6%)	24 (7.7%)	0.548
Antiplatelet agents, n (%)	34 (8.4%)	28 (8.9%)	0.790

* Indicates propafenone, amiodarone, or dronedarone. LBBAP = left bundle branch area pacing; RVP = right ventricular pacing; CAD = coronary artery disease; LBBB = left bundle branch block; RBBB = right bundle branch block; LAD = left atrial diameter; LVEDD = left ventricular end-diastolic diameter; LVEF = left ventricular ejection fraction; IVS = interventricular septum; MR = mitral regurgitation; TR = tricuspid regurgitation; AVB = atrioventricular block; SND = sinus node dysfunction; PM = pacemaker; ACEI/ARBs = angiotensin-converting enzyme inhibitors/angiotensin receptor blockers; CCB = calcium channel blocker; NOACs = novel oral anticoagulants.

**Table 2:** Comparison of pacing and procedural parameters in LBBAP and RVP groups.

Variables	LBBAP (*n* = 376)	RVP (*n* = 313)	*p* Value
LBB potential, n (%)	256 (68.1%)	-	-
P-V interval, ms	27.7 ± 4.7	-	-
Sti-LVAT at 5 V/0.4 ms, ms	73.9 ± 13.4	-	-
Sti-LVAT at 2 V/0.4 ms, ms	76.7 ± 15.4	-	-
Ring capture at 2 V/0.4 ms, n (%)	366 (97.3%)	-	-
Ring capture threshold, V/0.4 ms	1.04 ± 0.65	-	-
Capture threshold, V/0.4 ms	0.64 ± 0.22	0.64 ± 0.20	0.573
Paced QRSd, ms	114 ± 10.7	148 ± 18.0	<0.001
Pacing impedance, Ω	783 ± 154	782 ± 217	0.231
R wave amplitude, mV	11.7 ± 6.1	10.6 ± 4.9	0.142
Procedural duration, min	11.0 (7.0, 18.8)	6.7 (5.8, 7.8)	<0.001
Fluoroscopy duration, min	5.0 (3.0, 8.0)	2.8 (1.9, 3.5)	<0.001

LBBAP = left bundle branch area pacing; RVP = right ventricular pacing; LBB = left bundle branch; P-V interval = interval from LBB potential to ventricle; Sti-LVAT: pacing stimulus to left ventricular activation time; QRSd = QRS duration.

**Table 3:** Multiple linear regression analysis for the magnitude of delta left atrial diameter (ΔLAD).

Variables	β	95% CI	*p* Value
Age	0.045	0.003, 0.087	0.035
Female (vs. Male)	0.055	−1.062, 1.173	0.923
LBBAP (vs. RVP)	−1.601	−3.094, −0.109	0.036
Hypertension	0.429	−0.724, 1.581	0.465
Diabetes	−1.207	−2.613, 0.200	0.092
CAD	0.417	−1.060, 1.894	0.579
Atrial fibrillation	2.113	0.900, 3.325	0.001
Valvular heart disease	1.010	−0.907, 2.927	0.301
AVB	0.185	−1.433, 1.802	0.822
SND	−0.588	−1.880, 0.705	0.372
Device type (DDD vs VVI)	0.040	−0.941, 1.020	0.936
Baseline LAD	−0.433	−0.517, −0.349	<0.001
Baseline LVEDD	0.019	−0.073, 0.112	0.683
Baseline LVEF	−0.128	−0.216, −0.040	0.004
VP% ≥ 40%	0.116	−1.237, 1.469	0.866
Beta blockers	0.026	−0.006, 0.058	0.113
ACEI/ARBs	0.022	−0.006, 0.049	0.128
CCB	−0.247	−2.290, 1.795	0.812
Antiarrhythmic drugs *	−0.849	−2.299, 0.600	0.250

* Indicates propafenone, amiodarone, or dronedarone. LBBAP = left bundle branch area pacing; RVP = right ventricular pacing; CAD = coronary artery disease; AVB = atrioventricular block; SND = sinus nodal disfunction; LVEDD = left ventricular end-diastolic diameter; LVEF = left ventricular ejection fraction; VP% = percentage of ventricular pacing; ACEI/ARBs = angiotensin-converting enzyme inhibitors/angiotensin receptor blockers; CCB = calcium channel blocker.

**Table 4:** Procedure-related complications at implant and during follow-up.

Procedure-Related Complications	LBBAP (*n* = 376)	RVP (*n* = 313)
At implant		
Lead dislodgement, n (%)	1 (0.27%)	2 (0.64%)
Lead perforation during procedure, n (%)	1 (0.27%)	0 (0%)
Transient RBB injury, n (%)	30 (7.98%)	0 (0%)
Persistent RBB injury, n (%)	8 (2.13%)	0 (0%)
Pericardial effusion, n (%)	0 (0%)	0 (0%)
Pacing system infection, n (%)	0 (0%)	0 (0%)
Pocket hematoma, n (%)	0 (0%)	0 (0%)
Pneumothorax/hemothorax, n (%)	0 (0%)	0 (0%)
During follow-up		
Lead dislodgement, n (%)	0 (0%)	2 (0.64%)
Lead perforation, n (%)	0 (0%)	0 (0%)
Pocket hematoma, n (%)	0 (0%)	0 (0%)
Pacing threshold > 2.0 V/0.4 ms, n (%)	0 (0%)	0 (0%)
Pacing system infection, n (%)	0 (0%)	0 (0%)

## Data Availability

The data presented in this study are available on request from the corresponding author.
